# Role of the microbiota in inflammation-related related psychiatric disorders

**DOI:** 10.3389/fimmu.2025.1613027

**Published:** 2025-08-20

**Authors:** Liying Zhou, Qunhua Wu, Lin Jiang, Jiaoyu Rao, Jianlin Gao, Fang Zhao, Xiaokang Wang

**Affiliations:** ^1^ Department of Obstetrics and Gynecology, Shenzhen Longhua District Central Hospital, Shenzhen, China; ^2^ Department of Pharmacy, Shenzhen Longhua District Central Hospital, Shenzhen, China; ^3^ Department of Breast and Thyroid Surgery, Shenzhen Longhua District Central Hospital, Shenzhen, China

**Keywords:** gut microbiota, central nervous system, microglia, psychiatric disorders, neuroinflammation

## Abstract

The immune interactions within the gut–brain axis represent a critical etiological factor in psychiatric disorders. The gut microbiota and their metabolites serve as biological mediators that regulate neuroimmune activation and suppression in the central nervous system (CNS). During intestinal immune activation, pro-inflammatory cytokines (*e.g.*, IL-6, TNF-α) propagate to the CNS *via* compromised blood–brain barrier (BBB) integrity or vagal afferent fibers, disrupting neurotransmitter metabolism and inducing microglial hyperactivation, thereby exacerbating neuroinflammation. Microglia, the principal immune sentinels of the CNS, adopt a pro-inflammatory phenotype upon peripheral inflammatory signaling characterized by morphological transformations, excessive chemokine/cytokine production (*e.g.*, IL-1β, IL-6), and dysregulated neurotransmitter dynamics. These mechanisms are strongly implicated in neuropsychiatric conditions such as major depressive disorder, anxiety disorders, autism spectrum disorder, and schizophrenia. Emerging microbiota-targeted therapies, including probiotic interventions and fecal microbiota transplantation, demonstrate therapeutic potential by restoring tryptophan homeostasis and modulating systemic inflammation. This review synthesizes current evidence on the regulatory role of the gut microbiota in inflammation-related psychiatric disorders, specifically emphasizing the microbial modulation of neuroimmune crosstalk and neurotransmitter synthesis (*e.g.*, serotonin, dopamine). Mechanistic insights into microbial metabolites, such as short-chain fatty acids and tryptophan derivatives, are critically evaluated for their dual roles in psychiatric disorders. These findings advance a unified framework for managing psychiatric comorbidities through precision modulation of the gut–brain axis.

## 1 Introduction

The gut–brain axis orchestrates bidirectional communication between the gut microbiota and central nervous system (CNS) through integrated neural, immune, and endocrine pathways (1). Central to this interaction, microbial metabolites such as short-chain fatty acids (SCFAs) and tryptophan derivatives critically regulate neurotransmitter homeostasis (*e.g.*, serotonin [5-HT] and dopamine synthesis) and modulate neuroinflammatory cascades, thereby shaping mood, cognition, and behavior (2). This microbiota–gut–brain axis (MGBA) operates *via* dynamic cross-talk; specifically, gastrointestinal microbes influence neural plasticity and inflammatory cytokine release, whereas CNS-derived signals reciprocally reshape microbial composition and metabolic activity (3, 4).

Inflammation is a central mediator of gut–brain dysregulation (5). Emerging evidence highlights gut inflammation as a pivotal driver of neuropsychiatric pathology (6, 7). Intestinal-derived inflammatory cytokines (*e.g.*, IL-6, TNF-α) elevate cerebral glutamine levels and activate vagal signaling, concurrently increasing blood ammonia content *via* hepatic metabolism (8, 9). This cascade compromises blood–brain barrier (BBB) integrity. Subsequent BBB permeability facilitates the entry of cytokines and chemokines into the brain, in which they interact with neural receptors. These processes directly impair neuronal function through reductions in 5-HT, dopamine, and norepinephrine levels, thereby exacerbating inflammation-related psychiatric disorders (10, 11). These cytokines disrupt neurotransmitter metabolism, such as inhibiting tryptophan hydroxylase, thereby diverting tryptophan from 5-HT synthesis toward neurotoxic kynurenine derivatives, and induce microglial hyperactivation, amplifying neuroinflammation (12, 13). Microglia, constituting 5%–12% of brain cells, serve as the CNS’s primary immune sentinels (14). Upon activation by peripheral inflammatory signals, these cells adopt a pro-inflammatory phenotype characterized by morphological changes, chemokine/cytokine overproduction (*e.g.*, IL-1β, IL-6), and dysregulated neurotransmitter dynamics. This microglial dysfunction is mechanistically linked to neuropsychiatric disorders including depression (15), anxiety (16), autism (17), and schizophrenia (18). Critically, gut dysbiosis exacerbates this cycle by impairing BBB integrity and priming both peripheral and central immune systems for sustained cytokine release (2, 19).

Chronic inflammation is a transdiagnostic nexus in gut–brain disorders. Persistent inflammation has emerged as a shared pathogenic thread across gastrointestinal and psychiatric conditions. Notably, 38.9% of patients with inflammatory bowel disease (IBD) exhibit comorbid depression during active flares, with the prevalence of anxiety soaring to 80% (20). Even in remission, patients with IBD retain a 2–3-fold higher risk of depression/anxiety than the general population, with symptom severity correlating directly with intestinal inflammation intensity and disease chronicity (20). Mechanistically, IL-6, the levels of which are elevated systemically in IBD, permeates the BBB or relays signals *via* vagal pathways to suppress hippocampal neurogenesis and induce depression-like behaviors in preclinical models (21). Clinical validation of this axis is underscored by robust correlations between serum IL-6 levels and Hamilton Depression Rating Scale scores in IBD cohorts (22). These findings position gut-derived inflammation as both a biomarker and therapeutic target for neuropsychiatric comorbidities.

## 2 Gut microbial signatures in major psychiatric disorders

Growing evidence indicates that gut dysbiosis can influence brain function and contribute to neurological disorders *via* the gut–brain axis. Dysregulation of this axis is increasingly recognized as a pathophysiological basis for cognitive and psychiatric impairments (23). Consequently, targeted modulation of disrupted microbial ecosystems has emerged as a promising therapeutic strategy for such conditions. **Table 1** summarizes gut microbial alterations and inflammatory mediator profiles across major psychiatric disorders.

**Table 1:** Gut microbiota alterations and inflammatory mediator signatures in psychiatric disorders.

Psychiatric disorders	Microbiota changes	Metabolites and their synthetic pathway changes	Inflammatory factor regulation	References
Depression	↑ Firmicutes/Bacteroidetes Ratio, *Clostridium*, *Desulfovibrio, Bacteroides*, *Proteobacteria*, *Actinomycetes, Enterobacteriaceae, Mycobacteriaceae*, *Escherichia* ↓ *Blautia*, *Faecalibacterium*, *Bifidobacterium, Lactobacillus*,	↑ Propionic acid, isobutyric acid, isovaleric acid ↓ 5-HT, norepinephrine, butyric acid	↑ IL-6, TNF-α, caspase-1, IL-1β, CD8^+^ T cells ↓ IL-10	(24, 25, 57)
Anxiety disorders	↑ *Clostridium*, *Desulfovibrio*, *Lactobacillaceae*, Clostridia ↓ *Bifidobacterium*, *Akkermansia*	↑ p-Cresol ↓ Butyric acid	IL-6, TNF-α IL-10	(28–30)
Schizophrenia	↑ *Klebsiella*, *Clostridium difficile*, *Vibrio succinate*, *Proteobacteria*, *Lactobacillus*, *Streptococcus vestibularis* ↓ *Bifidobacterium*, *Prevotella*, *Blautia* spp., *Ruminococcus* spp., *Faecocci*, *Rossella*	↑ Kynurenine pathway activation ↓ Butyric acid, propionic acid, 5-HT, oleic acid, linolenic acid	↑ IL-18, IL-1Ra, TNF, IL-6	(35, 36, 58, 59)
BD	↑ *Streptococcus mitis*, *Streptococcus oralis*, *Streptococcus pseudopneumoniae*, *Fusobacterium varium*, *Fusobacterium* spp., *Urmitella timonensis*, *Bacteroides barnesiae*, *Bacteroides togonis*, *Bacteroidaceae* spp., *Actinomyces graevenitzii*, *Actinomyces oris*, *Actinomyces* spp., *Varibaculum cambriense* ↓ *Akkermansia muciniphila*, *Akkermansia* spp., *Yersinia aleksiciae*, *Acidaminococcus fermentans*, *Eubacterium eligens*, *Providencia alcalifaciens*, *Faecalibacterium prausnitzii*	↑ Vitamin B ↓ Aromatic amino acid, 2-hydroxybutyric acid, 3- methylpropionic acid, riboflavin, kynurenic acid	↑ IL-6, IL-1β, TNF-α, CRP ↓ IL-10	(39, 60)
PD	↑ *Akkermansia muciniphila*, *Prevotella* spp. (sp900313215), *Alistipes* spp., Rikenellaceae, *Bifidobacterium* spp., *Sphingomonas* spp., *Ruminococcaceae*, *Agathobacter* spp., *Gemmatimonas* spp. ↓ *Bacteroides fragilis*, *Anaerostipes hadrus*	↑ Butyrate synthesis ↓ S‐adenosylmethionine, fecal branched chain amino acids, aromatic amino acids	↑ LPS, IL-6, TNF-α IL-1β, ROS	(61–64)
AD	↑ *Escherichia coli*, *Clostridium*, Bacteroidetes, *Proteobacteria*, Verrucomicrobia ↓ *Bifidobacterium*, *Lactobacillus*, *Firmicutes*	↑ PUFA, arachidonic acid, phenylalanine, isoleucine ↓ Butyric acid, propionic acid	↑ IL-6, IL-1β, TNF-α, ROS	(47–49, 65)
ASD	↑ *Actinomyces*, *Aeromonas*, *Bacteroides*, *Corynebacterium*, *Clostridium*, *Desulfovibrio*, *Porphyromonas*, *Roseburia*, *Sutterella*, *Candida* spp. ↓ *Alistipes*, *Akkermansia*, *Bifidobacterium*, *Blautia*, *Collinsella*, *Enterococcus*, *Dialister*, *Faecalibacterium*, *Prausnitzii*, *Lactococcus*, *Prevotella*, *Staphylococcus*	↑ 4-Ethylphenyl sulfate, indolepyruvate, 5-HT, glycolate, imidazole propionate, N-acetylserine, p-cresol ↓ SCFAs, 5-aminovaleric acid, taurine	↑ CCL5, eotaxin, S100B, calprotectin, LPS, IL-2, IL-6, IL-12, TLR3 Signal pathway	(52, 54, 66–69)

↑, increased microbiota abundance and elevated concentrations of secretions or metabolites; ↓, decreased microbiota abundance and elevated concentrations of secretions or metabolites

### 2.1 Depression

In major depressive disorder (MDD), the most affected bacterial phyla include Firmicutes, Actinobacteria, and Bacteroidetes, leading to an elevated Bacteroidetes/Firmicutes ratio (24). Characteristic changes involve the enrichment of *Bacteroides* and depletion of *Blautia*, *Faecalibacterium*, and *Coprococcus*. In addition, an increased abundance of *Eggerthella* and reduced abundance of *Sutterella* are consistently observed in patients with MDD. A pathological vicious cycle is supported by evidence revealing an elevated abundance of pro-inflammatory genera (*e.g.*, *Escherichia*) in depression (25). These microbial shifts could drive MDD pathogenesis. Research on post-intervention outcomes of intestinal probiotics demonstrated that supplementation with Bre1025 (*Bifidobacterium longum*) restores 5-HT levels in the brain while concurrently suppressing serum corticosterone and pro-inflammatory cytokine expression (*e.g.*, IL-1β, IL-6) and elevating the expression of the anti-inflammatory cytokine IL-10 (26). Furthermore, JB-1 (*Lactobacillus rhamnosus*) supplementation alters gamma-aminobutyric acid (GABA) neurotransmission *via* the vagus nerve, thereby ameliorating depressive symptoms (27).

### 2.2 Anxiety disorders

Patients with anxiety disorders exhibit an increased Firmicutes/Bacteroidetes ratio, marked by the overgrowth of *Clostridium* and *Desulfovibrio* alongside reductions in the abundance of SCFA-producing genera (e.g., *Bifidobacterium*, *Lactobacillus*) (28). *Clostridium* species can exacerbate anxiety-like behaviors by generating neurotoxic metabolites (*e.g.*, p-cresol), which disrupt dopamine and 5-HT metabolism. Recent studies revealed distinct gut microbial community structures across anxiety states, with the abundance of *Akkermansia* being inversely correlated with anxiety severity (29, 30). *Akkermansia muciniphila* synergizes with lactate to restore the tryptophan metabolic balance, promoting 5-HT synthesis and alleviating anxiety (29).

### 2.3 Schizophrenia

Schizophrenia is associated with distinct gut microbiota alterations characterized by reduced α-diversity, featuring an increased abundance of *Proteobacteria* and *Lactobacillus* alongside diminished levels of anti-inflammatory commensals such as *Prevotella* (31). The condition exhibits a pro-inflammatory microbial profile with an increased abundance of *Lachnoclostridium* and a decreased abundance of SCFA-producing *Blautia* spp. and *Ruminococcus* spp (32)., corresponding with elevated lipopolysaccharide (LPS) (32) and reduced superoxide dismutase-1 levels (33). Notably, the abundance of *Lachnoclostridium* might predict poor cognitive improvement (34), whereas acute-phase patients display *Streptococcus vestibularis* enrichment associated with cognitive decline (35). Mechanistically, impaired SCFA synthesis (butyrate/propionate) compromises immunomodulation, and dysregulated tryptophan metabolism (*via* the kynurenine pathway) reduces 5-HT content, exacerbating the neurotransmitter imbalance (36). Gut dysbiosis further activates the vagus nerve–hypothalamic–pituitary–adrenal axis, inducing hippocampal microglial M1 polarization, whereas SCFA deficiency directly impairs neuronal mitochondrial function, amplifying oxidative stress (37). These findings collectively suggest that gut microbiota dysbiosis plays a multifaceted role in schizophrenia pathogenesis through immune–metabolic–neural pathways.

### 2.4 Bipolar disorder

Patients with BD exhibit gut dysbiosis, including disrupted *Firmicutes/Bacteroidetes* ratios, reduced α-diversity, and altered β-diversity. The elevated abundance of *Streptococcaceae* and *Bacteroidaceae* contrasts with the depletion of *A. muciniphila* and *F. prausnitzii* (38). Functional analyses revealed significant differences in amino acid metabolism and vitamin synthesis pathways (39). Notably, the abundance of *Faecalibacterium* (an anti-inflammatory, gram-positive commensal) is reduced in BD, IBD, and depression, suggesting gut dysbiosis can broadly disrupt CNS physiology.

### 2.5 Parkinson’s disease

PD is associated with gut microbial dysbiosis characterized by an increased abundance of *Lactobacillus* and *Bifidobacterium* but reduced levels of *Faecalibacterium*, *Coprococcus*, and *Blautia* (40, 41). Probiotic interventions (e.g., *L. casei*) mitigate β-amyloid deposition and cognitive decline, highlighting microbial metabolites as potential therapeutic targets (42, 43). Altered branched-chain and aromatic amino acid levels in fecal samples are correlated with PD progression (44, 45). Recent clinical studies indicated that patients with PD exhibit a reduced abundance of *Blautia* and diminished fecal levels of the SCFA butyrate. The abundance of *Blautia* is correlated with the clinical severity of PD. The RAS-related pathway, a pivotal inflammatory signaling pathway modulated by butyrate, has emerged as a key mechanism inhibiting microglial activation in PD. Alterations in the RAS–NF-κB pathway have been observed in patients with PD. Furthermore, butyrate derived from *B. producta* inhibited microglial activation by regulating the RAS–NF-κB pathway (46).

### 2.6 Alzheimer’s disease

The gut microbiota drives AD pathology through the “leaky gut–systemic inflammation–neuroimmune activation” axis, in which intestinal barrier dysfunction triggers systemic inflammation, which ultimately activates neuroimmune responses (47). In patients with AD, this process is characterized by reduced microbial diversity, with decreased levels of probiotics (*e.g.*, *Bifidobacterium*) and increased levels of opportunistic pathogens (*e.g.*, *E. coli*, *Clostridium*) and poly-unsaturated fatty acids (PUFAs), along with diminished levels of anti-inflammatory SCFAs, thereby exacerbating neuroinflammation (48, 49). Therapeutic strategies targeting this axis include probiotic supplementation (e.g., *Lactobacillus*) and high-fiber diets to boost SCFAs and suppress pro-inflammatory pathways, short-term antibiotic regimens or fecal microbiota transplantation (FMT), which has displayed efficacy in murine models (although long-term safety validation is required), and gut–brain axis-targeted drugs such as GV-971 that remodel the microbial balance while inhibiting neuroinflammation and improving cognitive function (50). These microbiota-modulating approaches represent promising strategies for delaying AD progression.

### 2.7 Autism spectrum disorder

ASD, a neurodevelopmental disorder marked by social deficits and repetitive behaviors, is linked to gut–brain crosstalk *via* microbial metabolites (51). Higher concentrations of p-cresol exhibit a significant link to increased symptom severity in ASD, demonstrating a strong correlation with both intensified behavioral symptoms and developmental regression patterns. P-cresol contributes to the pathogenesis of ASD by inducing dopamine accumulation and enhancing dopamine metabolism in the brain. This effect is partly explained by evidence identifying p-cresol as an inhibitor of dopamine β-hydroxylase (DBH)—the enzyme responsible for converting dopamine (DA) into norepinephrine (NE). By blocking dopamine’s transformation into norepinephrine, p-cresol further amplifies dopamine accumulation and promotes heightened dopaminergic metabolic activity within neural systems and reward circuitry (52, 53). Emerging evidence indicates that *Candida* spp. contribute to immune dysregulation, behavioral abnormalities, and alterations in brain activity, corroborated by their elevated prevalence in the feces of individuals with ASD. This genus might exacerbate hyperserotonemia through enhanced peripheral 5-HT production coupled with impaired brain 5-HT synthesis from tryptophan, thereby aggravating neurobehavioral symptoms (54). Biomarkers include brain-derived neurotrophic factor (BDNF), calprotectin, S100B, and dysregulated cytokines (*e.g.*, CCL5, eotaxin), underscoring the role of gut–microbiota–immune interactions in ASD pathogenesis (55, 56). Specific ASD-related microbial shifts and potential differential mechanisms are summarized in **Table 1**.

## 3 Gut-mediated mechanisms in inflammation-related psychiatric disorders

**Figure 1:**
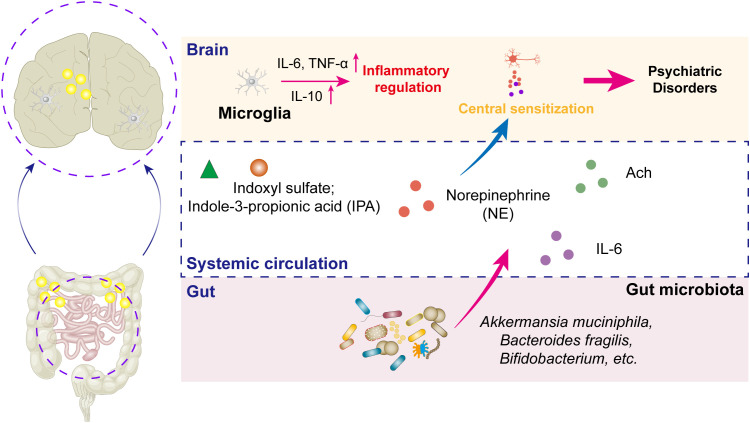
Schematic diagram of the molecular mechanisms underlying inflammation-related psychiatric disorders *via* the MGBA. Inflammation serves critical bridging roles within the MGBA, mediating the onset and progression of psychiatric disorders. Furthermore, the gut microbiota and their metabolites regulate norepinephrine secretion, which acts synergistically with acetylcholine (Ach) released from parasympathetic neurons to modulate central sensitization capacity within neural circuits, thereby significantly contributing to the neurobiological mechanisms of psychiatric pathologies.

The vagus nerve, serving as the principal bidirectional neural conduit between the gut and brain, mediates the transmission of inflammatory signals and metabolic information through its afferent and efferent fibers (70). This nerve constitutes a critical neuroanatomical bridge in gut–brain axis communication, directly regulating inflammatory-related psychiatric disorders *via* its synaptic connections to limbic structures (*e.g.*, hippocampus, amygdala) and hypothalamic nuclei (71). In parallel, the microbiota–immune–neural circuit interaction mechanism exhibits greater complexity in modulating inflammation-associated psychiatric conditions (72). This tripartite crosstalk involves microbial metabolite signaling (*e.g.*, SCFAs modulating microglial activation), neuroimmune synchronization (cytokine-mediated TLR4–NF-κB pathway activation), and enteroendocrine regulation (5-HT/dopamine synthesis influenced by gut microbes), as presented in **Figure 1**.

### 3.1 Bidirectional communication *via* the gut–brain axis

#### 3.1.1 Neural regulatory pathways

The vagus nerve serves as a direct neural conduit between the gut and brain, facilitating the bidirectional transmission of inflammatory signals and metabolic information (70). The vagus nerve facilitates the real-time gut-to-brain transmission of inflammatory signals and mechanical distension while enabling the brain-to-gut modulation of intestinal motility, secretion, and immune function *via* vagal efferents. Clinical evidence indicates that vagotomy increases the incidence of psychiatric disorders, whereas vagus nerve stimulation improves mood disorders (73, 74). Complementing this pathway, the enteric nervous system functions as an autonomous “second brain” through self-contained neuronal networks that process local reflexes independently while producing neurotransmitters such as dopamine and 5-HT to concurrently regulate gut functions (*e.g.*, peristalsis) and participate in mood modulation (47).

#### 3.1.2 Immune regulatory network

The disruption of gut microbiota homeostasis (dysbiosis) initiates systemic low-grade inflammatory responses via immune system activation, manifesting as an imbalance between elevated levels of pro-inflammatory cytokines (*e.g.*, IL-6, TNF-α) and deficient anti-inflammatory mediators (*e.g.*, IL-10) (75–77). These inflammatory signals cross the BBB or propagate through vagal afferents, subsequently activating microglial cells, exacerbating neuroinflammatory processes, and compromising prefrontal cortex-mediated executive functions including decision-making and emotional regulation (78). Recent mechanistic studies revealed that the gut microbiota directly influence neuroimmune crosstalk by modulating TRPV1-expressing sensory neurons (79). These neurons release calcitonin gene-related peptide, which orchestrates regulatory T cell (Treg) differentiation and functional activity, thereby maintaining the critical Th17/Treg equilibrium (80, 81). Furthermore, gut dysbiosis induces CD8^+^ T cell activation and the subsequent release of cytotoxic effector molecules (perforin and granzyme B), which in turn stimulate the colonic epithelium to produce the chemokine CXCL9, establishing a pro-inflammatory feedback loop (82).

Pro-inflammatory cytokines disrupt neurotransmitter metabolism (*e.g.*, inhibiting the conversion of tryptophan to 5-HT) and drive microglial activation, exacerbating neuroinflammation. Microglia play pivotal roles in neuroinflammatory cascades by releasing chemokines, cytokines, and reactive oxygen species (ROS) upon activation (83). Their phenotypic shift from surveillance to pro-inflammatory states disrupts neurotransmitter balance and synaptic plasticity.

Gut-derived LPS, a TLR4 ligand, activates NF-κB and MAPK pathways to promote cytokine release (75, 76, 84). In BD, elevated TLR4 expression in peripheral monocytes is correlated with disease severity (85). Targeting the gut microbiota (*e.g.*, probiotics) or TLR4 signaling might therefore ameliorate neuroinflammation in BD.

#### 3.1.3 Metabolite modulation

The gut microbiota profoundly influences emotional regulation through its production of key neurotransmitters such as 5-HT and dopamine that act *via* the gut–brain axis. Although only 5% of the body’s 5-HT originates in the brainstem, in which it regulates cognition, behavior, and metabolic functions as a neurotransmitter, the remaining 95% is synthesized peripherally in the gastrointestinal tract, in which it primarily functions as a vasoconstrictor and intestinal motility regulator (20, 54). Microbiota-derived SCFAs such as butyrate exert neuroprotective effects by inhibiting histone deacetylases and strengthening BBB integrity against inflammatory mediators. Importantly, gut microbes critically influence tryptophan metabolism. When preferentially shunted toward the kynurenine pathway rather than 5-HT synthesis, this process produces neurotoxic metabolites such as quinolinic acid that contribute to depression and cognitive impairment (86, 87). In mood disorders such as BP, circulating microbial metabolites (including SCFAs and LPS) can cross the BBB through vascular or neural pathways, disrupting monoaminergic neurotransmitter systems (5-HT and dopamine) and microglial activity, ultimately exacerbating mood instability. This multifaceted communication network highlights the gut microbiota’s central role in neuropsychiatric health through both direct neurotransmitter production and the indirect modulation of metabolic and immune pathways.

### 3.2 Microbiota–immune–neural circuit interactions

#### 3.2.1 Gut microbiota-mediated regulation of immunity

The gut microbiota modulate intestinal immunity through metabolites (SCFAs, tryptophan derivatives) and cell wall components (*e.g.*, LPS) (88). Key mechanisms include Treg expansion (Clostridia-derived butyrate induces dendritic cells to secrete TGF-β, promoting Treg differentiation) and Th17 suppression (Treg-secreted IL-10 inhibits Th17-mediated pro-inflammatory effects [*e.g.*, IL-17 release], maintaining immune homeostasis) (89).

#### 3.2.2 Immune–neuroinflammatory cascades

The peripheral immune status influences central neuroinflammation *via* three pathways. In the cytokine-to-brain axis, Th17/Treg imbalance elevates the expression of IL-6 and IL-1β, which cross the compromised BBB to activate microglia (90). Upon microglial polarization, pro-inflammatory cytokines drive M1 microglial polarization, releasing ROS and TNF-α to exacerbate neuronal damage (*e.g.*, synaptic pruning defects in autism) (90). Conversely, butyrate promotes M2 polarization *via* PPAR-γ activation, enhancing anti-inflammatory and reparative functions (88). Following BBB disruption, TNF-α upregulates matrix metalloproteinases, which degrade tight junction proteins (*e.g.*, occludin) and increase BBB permeability. This allows peripheral inflammatory mediators and microbial metabolites to infiltrate the brain parenchyma, inducing prefrontal cortical dysfunction (91).

In the amygdala, inflammatory mediators trigger microglial activation, leading to glutamatergic hyperexcitability and GABAergic synaptic impairment, which underlie anxiety-like behaviors and emotional dysregulation (92). Chronic neuroinflammation reduces synaptic plasticity in the prefrontal cortex and hippocampus, driving cognitive decline and mood disorders (93). Notably, butyrate enhances cognitive performance by modulating dorsal striatal activity (94).

#### 3.2.3 MGBA in psychiatric disorders

The MGBA mechanism in inflammation-related psychiatric disorders manifests as dysregulation across microbial, immune, and neural pathways, with detailed classifications and functional mechanisms presented in **Table 1**. In ASD and BD, microbiota–immune–neural dysregulation manifests as compositional shifts (reduced Bacteroidetes/Firmicutes ratio [*e.g.*, in ASD] decreases butyrate synthesis and increases inflammatory cytokines) (95, 96), pathogenic metabolites (Clostridia-derived p-cresol inhibits dopamine β-hydroxylase, blocking dopamine-to-norepinephrine conversion and inducing mood instability) (89), and tryptophan metabolism (microbiota-induced indoleamine 2,3-dioxygenase activation diverts tryptophan toward the kynurenine pathway, generating neurotoxic metabolites [*e.g.*, quinolinic acid]) (88).

## 4 Summary and perspectives

The widespread adoption of modern lifestyles has led to increasing tolerance to conventional pharmacotherapies in many patients. Future research should integrate multiomics datasets (metagenomic, metabolomic, and immunological profiling) to decipher the spatiotemporal dynamics of microbiota–host interactions. Personalized therapeutic strategies, particularly those leveraging microbiota signatures to predict anti-inflammatory treatment responses (e.g., stratified application of probiotics or anti-cytokine therapies), are emerging as critical frontiers. Furthermore, comparative analyses of microbiota-driven inflammatory signatures across psychiatric disorders might reveal transdiagnostic therapeutic targets, offering a unified approach for managing psychiatric comorbidities.

### 4.1 Microbiota-targeted therapies

Probiotic interventions demonstrate multifaceted therapeutic potential. Bifidobacterium enhances SCFA production, lowers intestinal pH to suppress pathogenic overgrowth, and upregulates BDNF to improve hippocampal neuroplasticity. L. casei mitigates cognitive decline by inhibiting β-amyloid aggregation and slowing disease progression in PD. F. prausnitzii, a keystone anti-inflammatory species, ameliorates CNS dysfunction in both IBD and depression. A. muciniphila synergizes with lactate to restore tryptophan metabolic balance, promoting 5-HT synthesis and alleviating anxiety.

FMT and probiotic formulations hold promise as alternatives to conventional pharmacotherapies, particularly in restoring tryptophan homeostasis and reducing peripheral IL-6 levels. Given their mechanistic versatility and safety profile, microbiota-targeted therapies are poised to gain clinical traction.

### 4.2 Synergistic anti-inflammatory drug applications

With more than 6.8 million patients globally affected by Crohn’s disease and ulcerative colitis, current therapies (e.g., immunosuppressants, biologics) remain palliative, often causing drug resistance and opportunistic infections with prolonged use (97). Notably, approximately 30% of patients with IBD develop anxiety or depression, underscoring the urgent need for dual-action therapies that concurrently resolve gut inflammation and modulate the gut–brain axis.

Preclinical evidence has highlighted the potential of synergistic strategies. Lamotrigine, a mood stabilizer, attenuates neuroinflammation by suppressing glutamatergic hyperactivity. Duloxetine, when co-administered with lamotrigine, potentiates 5-HT/norepinephrine reuptake inhibition, disrupting the inflammation–depression cycle. Such combinatorial approaches exemplify the paradigm shift toward targeting gut–brain axis dysregulation in psychiatric comorbidities. Future innovations could combine anti-inflammatory biologics (e.g., IL-6/IL-17 inhibitors) with neuromodulatory agents to achieve sustained remission.
